# Measuring the impact of seasonal malaria chemoprevention as part of routine malaria control in Kita, Mali

**DOI:** 10.1186/s12936-017-1974-x

**Published:** 2017-08-10

**Authors:** Fatou Diawara, Laura C. Steinhardt, Almahamoudou Mahamar, Tiangoua Traore, Daouda T. Kone, Halimatou Diawara, Beh Kamate, Diakalia Kone, Mouctar Diallo, Aboubacar Sadou, Jules Mihigo, Issaka Sagara, Abdoulaye A. Djimde, Erin Eckert, Alassane Dicko

**Affiliations:** 1 Malaria Research & Training Center, Faculty of Pharmacy and Faculty of Medicine and Dentistry, University of Sciences Techniques and Technologies of Bamako, P.O Box 1805, Bamako, Mali; 2 Malaria Branch, Centers for Disease Control and Prevention, 1600 Clifton Road NE, Mailstop A-06, Atlanta, GA 30333 USA; 3 Maternal and Child Survival Program, Save the Children, Bamako, Mali; 4 National Malaria Control Program, Rue: 108, Porte 106, Badalabougou, Bamako, Mali; 5 President’s Malaria Initiative-US Agency for International Development, P.O Box 34, Bamako, Mali; 6 President’s Malaria Initiative, USAID Bureau for Global Health, Office of Health, Infectious Diseases, and Nutrition, 2100 Crystal Drive, Arlington, VA 22202 USA

## Abstract

**Background:**

Seasonal malaria chemoprevention (SMC) is a new strategy recommended by WHO in areas of highly seasonal transmission in March 2012. Although randomized controlled trials (RCTs) have shown SMC to be highly effective, evidence and experience from routine implementation of SMC are limited.

**Methods:**

A non-randomized pragmatic trial with pre-post design was used, with one intervention district (Kita), where four rounds of SMC with sulfadoxine + amodiaquine (SP + AQ) took place in August–November 2014, and one comparison district (Bafoulabe). The primary aims were to evaluate SMC coverage and reductions in prevalence of malaria and anaemia when SMC is delivered through routine programmes using existing community health workers. Children aged 3–59 months from 15 selected localities per district, sampled with probability proportional to size, were surveyed and blood samples collected for malaria blood smears, haemoglobin (Hb) measurement, and molecular markers of drug resistance in two cross-sectional surveys, one before SMC (July 2014) and one after SMC (December 2014). Difference-in-differences regression models were used to assess and compare changes in malaria and anaemia in the intervention and comparison districts. Adherence and tolerability of SMC were assessed by cross-sectional surveys 4–7 days after each SMC round. Coverage of SMC was assessed in the post-SMC survey.

**Results:**

During round 1, 84% of targeted children received at least the first SMC dose, but coverage declined to 67% by round 4. Across the four treatment rounds, 54% of children received four complete SMC courses. Prevalence of parasitaemia was similar in intervention and comparison districts prior to SMC (23.4 vs 29.5%, p = 0.34) as was the prevalence of malaria illness (2.4 vs 1.9%, p = 0.75). After SMC, parasitaemia prevalence fell to 18% in the intervention district and increased to 46% in the comparison district [difference-in-differences (DD) OR = 0.35; 95% CI 0.20–0.60]. Prevalence of malaria illness fell to a greater degree in the intervention district versus the comparison district (DD OR = 0.20; 95% CI 0.04–0.94) and the same for moderate anaemia (Hb < 8 g/dL) (DD OR = 0.26, 95% CI 0.11–0.65). The frequency of the quintuple mutation (dhfr N51I, C59R and S108N + dhps A437G and K540E) remained low (5%) before and after intervention in both districts.

**Conclusions:**

Routine implementation of SMC in Mali substantially reduced malaria and anaemia, with reductions of similar magnitude to those seen in previous RCTs. Improving coverage could further strengthen SMC impact.

*Trial registration* clinical trial registration number NCT02894294

## Background

Malaria remains a major cause of morbidity and mortality in sub-Saharan Africa. The region is disproportionally affected by malaria, with 88% of the estimated 214 million malaria cases and 90% of 438,000 malaria deaths worldwide occurring in this region in 2015, according to recent World Health Organization (WHO) estimates. More than two-thirds (70%) of all malaria deaths occur in children under 5 years of age [1]. As in many countries in sub-Saharan Africa, malaria is the primary cause of morbidity and mortality in Mali, particularly among children under 5 years old. In 2015, the national health information system reported 2.4 million clinical cases of malaria in health facilities, accounting for 34% of all outpatient visits for all age groups. According to the 2015 Malaria Indicator Survey (MIS) [2] conducted during the high transmission period, the prevalence of parasitaemia among children 6–59 months of age was 32%.

Seasonal malaria chemoprevention (SMC) is a new strategy for malaria control recommended by WHO in March 2012 for sub-Saharan countries in the Sahel region. It involves the administration of therapeutic doses of anti-malarials [amodiaquine (AQ) + sulfadoxine–pyrimethamine (SP)] at monthly intervals during the high malaria transmission season. Specifically, the WHO recommends SMC at monthly intervals for up to 4 months per season for children aged 3–59 months living in highly seasonal transmission areas in the Sahel in West Africa where a high level of resistance to AQ or SP has not been recorded [3]. WHO does not specify the programming aspects except that it should be given using existing community platforms [4].

The Mali National Malaria Control Programme adopted SMC as policy in 2012 with a plan to expand implementation to all districts by 2016. Clinical trials conducted in Mali and Burkina Faso have shown a strong protective effect of SMC [5, 6]. In Mali, SMC reduced parasitaemia and clinical malaria (both uncomplicated and complicated) by over 80% in a trial setting [5]. Taken together, trials in Mali, Senegal and Burkina Faso showed that SMC reduced clinical malaria episodes by 82% (95% Confidence Interval (CI) [64; 87%], p < 0.001) [7].

Current evidence on SMC comes mainly from randomized control trials. Recent modelling studies [8–10] indicate a strong effect of SMC, suggesting that it is a promising intervention, but limited evidence from routine implementation has been reported. This study aims to evaluate SMC coverage and reductions in malaria and anemia when SMC is delivered through routine programmes by existing community health workers.

## Methods

### Study sites

Seasonal malaria chemoprevention was implemented in Kita district in 2014. Kita is located approximately 180 km north of the capital Bamako in the western Kayes Region. The health infrastructure includes a district hospital and 47 community health centres providing primary health, including curative, preventive, social, and promotional services for a catchment area of up 15 km, usually composed of several villages or quartiers. At the village level, community health centres are supported by community health workers who are members of the community chosen by community and who have received shorter training than professional workers to provide basic mother and child health services. In 2014, the district population was estimated at 516,649 inhabitants with about 77,497 children aged 3–59 months in 336 villages/quartiers. Bafoulabe, a neighbouring district in the same Kayes Region, with highly seasonal rainfall and malaria transmission similar to Kita, was chosen as the comparison district because it is adjacent to Kita, with similar geographic, social and demographic characteristics. In 2014 the rainfall was about 854 mm in the area of Bafoulabe and 839 mm in Kita. SMC was not implemented in Bafoulabe in 2014 due to funding constraints. Bafoulabe has a district hospital and 24 community health centres; in 2014 the population was estimated at 152,976 inhabitants with 22,946 children aged 3–59 months in 336 villages/quartiers. In 2013, parasitaemia prevalence among children ages 6–59 months in the Kayes Region, where Bafoulabe and Kita are located, was 36.9% according to the recent Demographic and Health Survey [11]. Both districts received a universal bed net campaign in April 2014 (one insecticide-treated bed net per two people) and it was expected that levels of bed net coverage would be similar in the two districts. Indoor residual spraying was not done in any of the two districts nor in other districts in the Kayes Region.

### Study design

A non-randomized, pre-post design was used, with an intervention district (Kita) where SMC was implemented through the health system, and a comparison district (Bafoulabe) where SMC was not implemented. SMC implementation consisted of the administration of SP + AQ at monthly intervals in children 3–59 months in August, September, October, and November 2014. During each round, children aged 3–11 months received 75 mg of AQ given once daily for 3 days plus a single dose of 250/12.5 mg of SP, while children aged 12–59 months received 150 mg AQ base given once daily for 3 days and a single dose of 500/25 mg of SP. The single dose of SP was given only on the first day, at the same time as the first dose of AQ. Tablets were crushed into water and sugar added and administered under direct observation by the health care worker. Children were observed for 30 min after drug administration, and drugs were re-administered if vomiting occurred during this period. The first day of treatment (SP + AQ) was administrated by health workers at fixed points in the village (health centres where possible or at a central point in villages where there was no health centre). The doses of AQ for the second and third days were given to parents to be administrated at home. The administration of the first day’s treatment was recorded on a special SMC card that was given to all children receiving SMC during the first round. The SMC implementation in Kita was carried out by health staff in the district, including 588 heath workers (nurses and community health workers) organized into 133 teams of 2–6 health workers each; 37 teams were based in health centres and 96 teams were deployed to fixed distribution sites in surrounding villages. The same locations were used at each of the four SMC rounds. Children between 3 and 59 months of age are eligible to receive SMC if they are afebrile, have no history of allergy to SP or AQ, not under co-trimoxazole prophylaxis for HIV infection or exposure.

Seasonal malaria chemoprevention activities were funded by the US President’s Malaria Initiative (PMI) through the Maternal and Child Survival Programme (MCSP) of the Save the Children. The National Malaria Control Programme and regional and national health directorates, in collaboration with PMI and Save the Children, led the planning and implementation of SMC. A research team of the Malaria Research and Training Centre of the University of Science Techniques and Technologies of Bamako that was not involved in the implementation conducted the surveys.

### Assessment of the impact of SMC on malaria, anaemia and molecular markers of the resistance to SP and AQ

Baseline and follow-up, cross-sectional, household surveys were carried out in the intervention and comparison districts to assess the impact of SMC on malaria parasitaemia, fever, malaria illness, and anaemia. The baseline survey was performed in July 2014 prior to the start of SMC implementation and the post-intervention (follow-up) survey took place in December 2014. Thirty clusters (villages or quartiers), 15 in each district, were selected using a random sampling with probability proportional to the size of the population. In each cluster, a sample of about 38 children aged 3–59 months were randomly selected and surveyed using the WHO EPI method [12]. Households were selected first and a child in target age group was selected from each of these households. For better comparability the pre-and post-surveys were conducted in the same localities (clusters) were used for the two surveys. After informed consent was obtained, a brief clinical examination (including history of fever and temperature measurement) was performed and a finger prick blood sample was collected for a blood smear, rapid diagnostic test (RDT) (in case of fever or symptoms suggestive of malaria), measurement of haemoglobin concentration, and filter paper for molecular markers of SP and AQ resistance. Children with a positive RDT were treated with artemether–lumefantrine, in line with current national case management guidelines. Children with moderate severe anaemia (haemoglobin < 8 g/dL) or with signs of severe malaria were referred to the nearest health centre for management. Malaria infection was defined as presence of malaria parasitaemia by blood smear. Fever was defined as axillary temperature ≥37.5 °C, and malaria illness was defined as fever and presence of asexual malaria parasitaemia by blood smear.

### Assessment of adherence to SMC and frequency of adverse events

Cross-sectional surveys were carried out 4–7 days after each of the four rounds of SMC to assess caregivers’ adherence to the administration of SMC drugs and determine the frequency of adverse events in the intervention district of Kita. Adherence surveys were conducted in 10 clusters (localities) selected randomly among the 15 already selected for the pre- post-intervention surveys. The same localities (clusters) but different households were surveyed after each round. The main indicators assessed through caregiver interview and verification of the SMC card included: the proportion of children who received the second and third dose of AQ at home, the proportion who spat out or vomited the treatment, the nature and frequency of the adverse events, and opinions of parents/caregivers regarding the intervention.

### Assessment of SMC coverage

During the post-SMC survey in Kita, 15 additional clusters (villages/quartiers) were added to the 15 surveyed at baseline, yielding a total of 30 clusters for the coverage survey in Kita. Parents or guardians of children in the target age range at the time of the SMC administration were questioned using standardized questionnaires about receipt of SMC. In addition, information from SMC cards was recorded for each child, when available. Interviews in each household were conducted by two trained interviewers in the local language. Primary indicators assessed from the coverage survey included administration of each day of the 3-day SMC treatment course during each of the four rounds and the use of insecticide-impregnated bed nets (ITN) during the previous night. Completed questionnaires were verified at the end of each day by a supervisor and corrected if necessary. Coverage of SMC at each round was defined as the proportion of the children aged 3–59 months at the time of SMC who received the 3 days’ treatment of SMC during that specific round. Full SMC coverage was defined as the proportion of children aged 3–59 months who received the complete 3-day treatment course during all four rounds of SMC.

### Impact on malaria morbidity using health records data

The study team initially intended to use routine data reported from health centres in the intervention and the comparison districts to assess trends in malaria cases, but these data were too incomplete. For example, data from several weeks (up to 3 months) were not reported for several health centres. For this reason, the impact on malaria morbidity was assessed using routine data on confirmed malaria cases extracted from the registers by the research team in nine of the 47 community health centres in Kita and 7 of the 24 health centres in Bafoulabe; these health centres were randomly selected from among the 15 health areas of pre- post-intervention surveyed in each of the two districts. These data on confirmed malaria, uncomplicated and severe cases for 2013 and 2014 were collected from registers in February 2015.

### Laboratory analysis

#### Rapid diagnostic test (RDT)

Malaria RDTs were performed in the field according to the manufacturers’ recommendation using the same RDTs used in the primary health centres (SD Bioline *Plasmodium falciparum*). These RDTs can detect infection with *Plasmodium* by detecting histidine-rich protein 2 antigen specific to *P. falciparum* (PfHRP2). RDT results were given to caregivers of children who were tested; those testing positive received malaria treatment according to national guidelines in Mali.

#### Haemoglobin measurement

HemoCue^®^ machines (Angelholm, Sweden) were used to measure haemoglobin (Hb) concentration in children using capillary blood obtained by finger prick.

#### Thick and thin blood smears

Thick and thin blood smears were prepared in the field for participating children in the pre-post intervention surveys. Thin smears were fixed in methanol. All slides were air dried and stained using Giemsa stain 3% for 45–60 min the field, and transferred to the Malaria Research and Training Centre (MRTC) laboratory for reading and storage. The stained thick and thin blood films were examined microscopically at a magnification of 1000× to identify the parasite species and to determine the parasite density. Parasite density was assessed by experienced certified microscopists unaware of the intervention and comparison groups, by counting the number of asexual parasites in a set number of white blood cells (typically 200) with a hand tally counter according to established standard operating procedures, assuming 8000 white blood cells (WBC)/µL.

#### Dried blood spots and polymerase chain reaction

Filter paper samples were air dried and stored individually in small Ziploc bags with a desiccant and transferred to the MRTC laboratory in Bamako for analysis. Molecular analysis was performed on samples from children whose blood smear showed *P. falciparum* parasitaemia ≥160/uL (4 parasites/200 WBC) by blood smear. Samples were analysed by nested polymerase chain reaction (PCR) and/or PCR-restriction fragment length polymorphism (RFLP) for mutations at codons 51, 59 and 108 of the *dhfr* gene, 437 and 540 of the *dhps* gene, mutations at codon 76 in the *P. falciparum* chloroquine transporter gene (*pfcrt*), and at codon 86 of the *P. falciparum* multidrug resistance gene one (*pfmdr1*) according to published methods [13, 14]. Cases of mixed infection (wild type and mutant) were categorized as mutant. Quintuple mutant was defined as the presence of the three dhfr mutations (N51I, C59R and S108N) and the two dhps mutations (A437G and K540E).

### Sample size

#### Household survey

Based on a meta-analysis of SMC that found a reduction of parasitaemia prevalence of 53% in trial settings [7], and assuming a 40% reduction under programmatic conditions for this study. A sample size of 556 children per district (total of 1112 children) was required to detect a 40% difference in parasitaemia between the SMC and non-SMC districts at follow-up (15 and 25% parasitaemia, respectively), with an alpha = 0.05, power of 80%, a design effect of 2.0, and 10% non-response.

#### Adherence surveys

The adherence surveys were powered to estimate an adherence rate (completing all three doses of SP − AQ) of 80%, within a 95% confidence limit of ±10%. Assuming a design effect of 2 and a 10% non-response rate, 156 children were required after each round. To achieve this sample size, 10 clusters were selected in Kita, and 20 households per cluster were visited and surveyed after each SMC round.

#### Coverage survey

Assuming a SMC coverage of 50%, with an alpha of 5%, design effect of 2 and non-response rate of up to 10%, a total of 840 children were required for the coverage survey. To achieve this sample size, 30 clusters were randomly selected in Kita and 38 children were surveyed in each cluster.

### Management and analysis of data

Survey data were collected on standardized forms, entered into a MS Access database, and analysed using Stata (version 12.1). As described above, data from health facility registers were extracted from the registers in selected heath centres in intervention and comparison districts. Analyses from household surveys were adjusted for cluster sampling, using the survey commands in Stata. Logistic regression was used for binary outcomes of anaemia and parasitaemia, and used a difference-in-differences approach, which can be useful in estimating impact from observational study designs [15]. These regression analyses were adjusted for potential confounding variables, age, gender, ITN use, and the cluster design. The level of significance was set at 5%.

## Results

### Coverage of SMC

The coverage survey was completed for 1141 children aged 4–63 months in December 2014 (children aged of 3 month at least at the time of the last round of SMC in November and did not reach the fifth anniversary at the time of the first round of SMC in August). The SMC card was available for 805 (70.6%) of 1141 children. Based on information collected by interview, the proportion of children who received at least the first day of treatment administered by the health worker was 84% at the first SMC round, but declined with subsequent rounds and was 67% for the fourth round (Table 1). The same trend was observed when the information collected on SMC card was used. The proportion of children who received all 3 days of treatment for all four rounds was 53.4%, according to caregiver interview. The reasons for not receiving SMC during the different rounds are summarized in Table 2. Travel during the SMC round accounted for 434 (43%) of 1007 missed visits and was the most frequent reason reported for not receiving SMC followed by parents reporting they were not informed about the SMC round [342 (38%) of 1007].

**Table 1:** Coverage of SMC defined as proportion of children who received SMC drugs at day 1 and days 1–3 according to the source of the information (interview or SMC card) during the coverage survey

	Interview^a^			SMC card^b^		
	n/N	%	95% CI	n/N	%	95% CI
Received at least the first day treatment of SMC at:						
Round 1	896/1170	83.7	75.6–91.9	664/766	86.7	82.0–91.3
Round 2	903/1101	82.0	74.7–89.3	610/790	77.2	68.7–85.7
Round 3	845/1116	75.7	67.6–83.8	523/797	65.6	55.4–75.8
Round 4	752/1118	67.3	59.0–75.5	463/796	58.2	46.3–70.0
Rounds 1, 2, 3 and 4	563/1046	53.8	44.5–63.1	309/760	40.7	29.2–52.1
Received all 3 days’ treatment of SMC at:						
Round 1	811/1068	75.9	67.4–84.7	–	–	
Round 2	900/1101	81.7	74.2–89.3	–	–	
Round 3	841/1116	75.4	67.3–83.4	–	–	
Round 4	747/1118	66.8	58.5–75.1	–	–	
Rounds 1, 2, 3 and 4	559/1046	53.4	44.1–62.7	–	–	

^a^Based on information from interview only

^b^Based on information on SMC card

**Table 2:** Reasons for not receiving SMC during the different rounds based on the interview in the district of Kita

Reasons	Round 1		Round 2		Round 3		Round 4		Total	
	n/N	%	n/N	%	n/N	%	n/N	%	n/N	%
Travel	58/174	33.4	79/197	40.3	124/271	42.1	183/365	50.2	434/1.007	43.1
Parents not informed	88/174	50.6	75/197	38.1	97/271	35.8	122/365	33.4	382/1.007	37.9
Refusal	9/174	5.2	21/197	10.7	29/271	10.7	25/365	6.8	84/1.007	8.3
Forgot	3/174	1.7	5/197	2.5	15/271	5.5	17/365	4.7	40/1.007	4.0
Others^a^	16/174	9.2	17/197	8.6	16/271	5.9	18/365	4.9	67/1.007	6.7

^a^Others: mother was sick, age <3 months, child was sick, mother was late, malaria treatment, carte not available, mother was busy, mother was absent, did not see the health worker, health worker refused to give SMC because parent left the SMC card at home

### Adherence and tolerance of SMC

Adherence and adverse event surveys were carried out in a total of 911 children across the four rounds. A child’s mother was the respondent during the interview more than 90% of the time (Table 3). Of children given the first dose of SMC by a health worker, 97.5% (811/832) were reported to have received the second day’s treatment by the caregiver, and 94.8% (789/832) the third day’s treatment for all rounds combined (Table 3). ‘Forgetting’ was the main reason reported for failing to administer the second and third day treatments, in 86% (18/21) and 74% (32/43), respectively. Among the parents of children who did not receive the third day treatment, one (2.3%) reported that the drug was given to another child and another parent (2.3%) reported that the drug was saved for future treatment in case the child gets sick.

**Table 3:** Persons interviewed in the household and administration of day 2 and day 3 treatments by the parents at home during the different SMC rounds, according to the adherence surveys

	Round 1		Round 2		Round 3		Round 4		Total	
	n/N	%	n/N	%	n/N	%	n/N	%	n/N	%
Person interviewed										
Father	6/230	2.6	5/231	2.2	3/225	1.3	5/225	2.2	19/911	2.1
Mother	201/230	87.4	212/231	91.8	213/225	94.7	203/225	90.2	829/911	91.0
Guardian	10/230	4.3	10/231	4.3	4/225	1.7	5/225	2.2	29/911	3.2
Others^a^	13/230	5.6	4/231	1.7	5/225	2.2	12/225	5.3	34/911	3.7
Administration of days 2 and 3 treatments at home										
Second day	209/211	99.0	197/206	95.6	197/202	97.5	208/213	97.6	811/832	97.5
Third day	205/211	97.2	191/206	92.7	186/202	92.8	207/213	97.2	789/832	94.8

^a^Grandfather, grandmother, brother, sister, uncle aunt, uncle, aunt

A total of 21% (177/729) of children were reported to have spat out or vomited the first day’s treatment, while 6.2% (50/808) and 6.6% (52/792) did so on days 2 and 3, respectively (Table 4). Overall, SMC drugs were well tolerated (Table 5). The most commonly reported adverse events were diarrhoea (7.8%) and vomiting (4.9%). Itching was reported in 1.7% of the children.

**Table 4:** Proportion of children who spat out/vomited SMC drugs by treatment day and round in Kita district, according to the adherence surveys

	Round 1		Round 2		Round 3		Round 4		Total	
	n/N	%	n/N	%	n/N	%	n/N	%	n/N	%
Day 1 spit out or vomited	57/210	27.1	34/204	16.7	32/202	15.8	54/213	25.3	177/829	21.3
Day 1 replacement dose given	11/56	19.6	18/33	54.5	14/32	43.5	32/54	40.7	75/175	42.9
Day 1 replacement dose spit out or vomited	2/11	18.2	0/18	0.0	0/14	0.0	4/32	12.5	6/75	8.0
Day 2 spit out or vomited	21/208	10.1	9/196	4.6	8/197	4.1	12/195	5.8	50/808	6.2
Day 2 replacement dose spitted or vomited	1/21	4.8	2/9	22.2	0/8	0.0	1/12	8.3	4/50	8.0
Day 3 spit out or vomited	17/210	8.1	9/189	4.8	12/186	6.4	14/207	6.8	52/792	6.6
Day 3 replacement dose spit out or vomited	0/17	0.0	0/9	0.0	0/12	0.0	0/13	7.1	1/51	1.9

**Table 5:** Adverse events reported in children after administration of SMC drugs in the district of Kita during the adherence surveys

Adverse events^a^	Round 1		Round 2		Round 3		Round 4		Total	
	n/N	%	(n = 206)		(n = 202)		(n = 212)		(n = 831)	
Diarrhea	15/211	7.1	14/206	6.8	19/202	9.4	17/212	8.0	65/831	7.8
Vomiting	15/211	7.1	5/206	2.4	11/202	5.4	10/212	4.7	41/831	4.9
Fatigue/weakness	2/211	0.9	2/206	1.0	6/202	3.0	3/212	1.4	13/831	1.6
Abdominal pain	1/211	0.5	2/206	1.0	1/202	0.5	1/212	0.5	5/831	0.6
Itching	1/211	0.5	5/206	2.4	6/202	3.0	2/212	0.9	14/831	1.7
Jaundice	1/211	0.5	2/206	1.0	4/202	2.0	0/212	0.0	7/831	0.8
Headaches	0/211	0.0	0/206	0.0	0/202	0.0	1/212	0.5	1/831	0.1
Urine discoloration	0/211	0.0	1/206	0.5	0/202	0.0	0/212	0.0	1/831	0.1

^a^A child can have more than one adverse event

### Parents’ opinion on SMC

Overall, 99.9% of parents of children surveyed reported that SMC is good or very good, with 68.3% saying it is very good (Table 6); 60% of parents reported that SMC prevents or protects against malaria and 35% reported that it improves health. Of the 901 parents who gave their opinion, all except one (99.9%) requested that SMC be continued.

**Table 6:** Parents’ opinion on SMC and justification of the opinion in the district of Kita during the adherence surveys

	Round 1		Round 2		Round 3		Round 4		Total	
	n/N	%	n/N	%	n/N	%	n/N	%	n/N	%
Parent opinion on SMC										
Very good	153/229	66.8	156/227	68.7	156/224	69.6	149/218	68.3	614/898	68.4
Good	75/229	32.8	71/227	31.3	68/224	30.4	69/218	31.7	283/898	31.5
Bad	1/229	0.4	0/227	0.0	0/224	0.0	0/218	0.0	1/898	0.1
Justification of the opinion										
Improve health	57/229	24.9	79/227	34.8	74/224	33.0	103/218	47.2	313/898	34.8
Prevent malaria	114/229	49.8	72/227	31.7	88/224	39.3	52/218	23.9	326/898	36.3
Fight malaria	57/229	24.9	63/227	27.6	49/224	21.8	48/218	21.3	317/898	23.9
Save money	0/229	0.0	7/227	3.0	8/224	3.6	8/218	3.6	23/898	2.5
Ensure good health	0/229	0.0	6/227	2.6	4/224	1.8	7/218	3.1	17/898	1.9
No harmful effect	0/229	0.0	0/227	0.0	1/224	0.4	0/218	0.0	1/898	0.1
Calm the mothers	1/229	0.4	0/227	0.0	0/224	0.0	0/218	0.0	1/898	0.1

### Prevalence of malaria infection disease and anaemia in pre and post-intervention surveys

The baseline survey on malaria and anemia before the implementation of SMC was carried out in 589 children aged 3–59 months in Kita and 573 in Bafoulabe. The post-implementation survey included 573 children Kita and 576 children in Bafoulabe. The gender distribution was similar in the two districts for both surveys. At baseline, 52% of children in Kita and 54% in Bafoulabe were male, p = 0.46. These proportions in post-intervention survey were 48 and 52%, respectively, in Kita and Bafoulabe; p = 0.11. Mean age was not significantly different between the two districts both at baseline (26.7 months in Kita vs 27.4 months in Bafoulabe p = 0.64) and in the post-intervention survey (29.4 months in Kita vs 28.6 months in Bafoulabe p = 0.47).

**Fig. 1:**
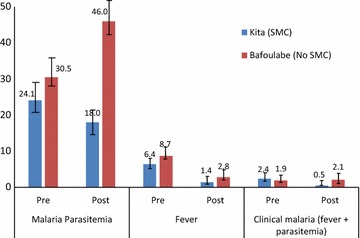
Proportion of children aged 3–59 months with malaria parasitaemia, fever and clinical malaria pre- and post-SMC in the intervention district of Kita in 2014

The prevalence of malaria infection aged 3–59 months was similar in the two districts at baseline (24.1% in Kita vs 30.5% in Bafoulabe p = 0.32) (Fig. 1). At the end of the intervention period, the prevalence of malaria infection was 46% in the comparison district of Bafoulabe compared to 18% in the intervention district of Kita, corresponding to a reduction of 65% greater reduction in malaria infection prevalence in the intervention district compared to the comparison district (difference-in-differences OR = 0.35, 95% CI 0.19–0.66), p < 0.001.

The prevalence of fever (defined as an axillary temperature ≥37.5 °C) was similar across the two districts at baseline (6.4 vs 8.7%; p = 0.37). In post-intervention period, the prevalence of fever was twice as less in the intervention district as in the comparison district but the difference was not statistically significant (2.8 vs 1.4%); p = 0.11) (Fig. 1). Prevalence of malaria illness defined as axillary temperature ≥37.5 °C and the presence of asexual forms of malaria parasites at blood smear was also similar in the two districts at baseline (2.4 vs 1.9%, p = 0.75). At the end of the intervention period, the prevalence of malaria illness was significantly lower in the intervention area than in the comparison area (0.5 vs 2.1%) corresponding to 80 and 80% greater reduction in the prevalence of malaria illness pre and post in the intervention district compared to comparison district (difference-in differences OR = 0.20; 95% CI 0.04–0.94; p = 0.04) adjusted for age, gender and cluster.

**Fig. 2:**
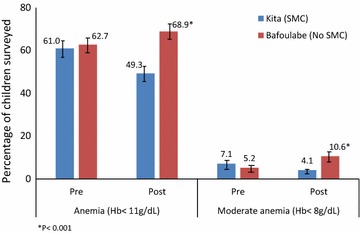
Percentage of children aged 3–59 months with any anaemia and moderate anaemia pre- and post-SMC, in the intervention district of Kita in 2014

The prevalence of anaemia was similar in the intervention and comparison districts prior to the intervention (61.0 vs 62.7%, p = 0.73). At the end of the intervention period, the prevalence of anaemia in children aged 3–59 months was 68.9% in the comparison district and 49.3% in the intervention district, corresponding to a reduction in odds of anemia of 53% in the intervention area (difference-in differences OR = 0.47, 95% CI 0.26–0.87, p = 0.019) adjusted for age, gender, ITN use and cluster design (Fig. 2). The same trends were observed for moderate anaemia with 74% reduction of the odds in the intervention district (difference-in differences OR = 0.26, 95% CI 0.11–0.65, p = 0.005) adjusted for age, gender, ITN use, and cluster. There were no cases of severe anaemia (defined as haemoglobin <5 g/dL) at either time point.

The proportions of children aged 3–59 months sleeping under ITNs were high and similar in the comparison and intervention districts in pre-intervention survey (99.1 vs 99.7%, p = 0.27) as well as in post-intervention survey (97.1 vs 98.8% p = 0.14%).

### Molecular markers of resistance to SP and AQ

The frequencies of individual and multiple mutations in *dhfr*, *dhps*, *Pfcrt*-76T and *Pfmdr1*-86Y were similar between the two districts before and after the intervention except for single mutation *dhps* A437G, which was higher in the intervention district compared to the comparison district at the end of the intervention (p = 0.01) (Table 7). The level of *dhps* K540E mutation (12.5% at the highest) was well below the 50% limit recommended by WHO for not using the SP in intermittent preventive treatment in infants and did not vary before and after intervention in the two districts. There was also no significant change in prevalence of the quintuple mutants before and after the intervention and between the two districts.

**Table 7:** Frequency of molecular markers associated with resistance of *Plasmodium falciparum* to SP and AQ in the districts of Kita and Bafoulabe during the pre- and post-intervention surveys

	Kita		Bafoulabe		p
	n/N	%	n	%	
Pre-intervention (July 2014)					
dhfrN51I	38/48	79.2	50/58	86.2	0.42
dhfrC59R	39/48	81.2	54/58	93.1	0.07
dhfrS108 N	42/48	87.5	53/58	91.4	0.59
Triple dhfr (N51I, C59R, S108N)	37/48	77.1	49/58	84.8	0.45
dhps A437G	20/48	41.8	23/56	42.0	0.95
dhps K540E	6/48	12.5	2/58	3.4	0.11
Quintiple (triple dhfr+ dhpsA437G et K540E)	2/48	4.2	1/56	1.8	0.41
Pfmdr1-86Y	8/42	19.0	13/54	24.1	0.60
Pfcrt-76T	44/48	91.7	51/57	89.5	0.25
Post intervention (December 2014)					
dhfrN51I	38/38	100	79/79	100	1
dhfrC59R	38/38	100	77/79	97.5	0.46
dhfrS108N	37/38	97.4	68/78	87.2	0.10
Triple dhfr (N51I, C59R, S108N)	37/38	97.4	66/78	87.2	0.10
dhps A437G	25/38	65.8	35/79	44.3	0.01
dhps K540E	1/38	2.6	5/79	6.3	0.47
Quintuple (triple dhfr+ dhpsA437G et K540E)	1/38	2.6	1/78	1.8	0.60
Pfmdr1-86Y	7/38	18.4	8/79	10.1	0.60
Pfcrt-76T	28/38	73.7	59/78	75.6	0.81

### Malaria morbidity based on routine data

**Fig. 3:**
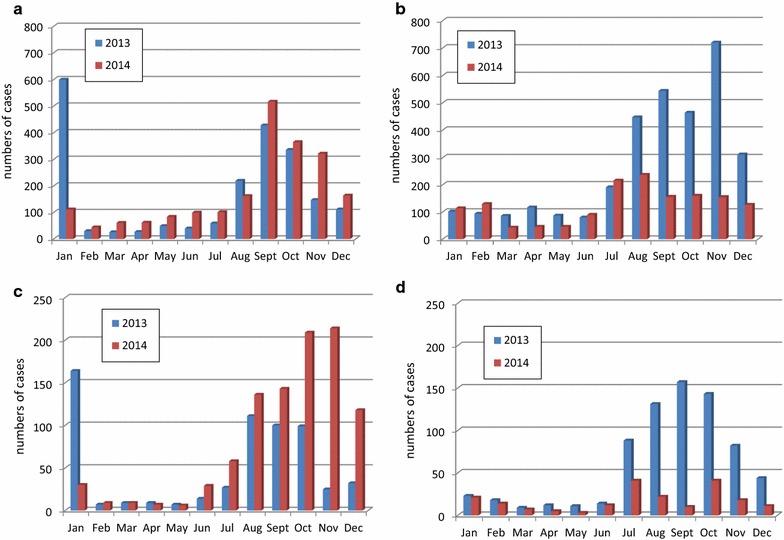
Numbers of confirmed uncomplicated and severe malaria cases in children under five in nine selected health centres in the intervention district of Kita and seven in the comparison district of Bafoulabe in 2013 and 2014. **a** Confirmed cases of malaria in Bafoulabe (comparison district); **b** confirmed cases of malaria in Kita (intervention district); **c** confirmed cases of severe malaria in Bafoulabe (comparison district); **d** confirmed cases of severe malaria in Kita (intervention district)

In the localities surveyed in Kita, the number of confirmed malaria cases during the 2014 transmission season (July to December) was reduced by 61% compared to 2013, whereas in the comparison district of Bafoulabe there was an increase of 23% (Fig. 3). The number of severe malaria cases was also reduced by 72% in the intervention district of Kita during the 2014 transmission season compared to 2013 season, whereas in the comparison district of Bafoulabe, the number more than doubled (increased by 123%) in 2014 compared 2013.

## Discussion

Seasonal malaria chemoprevention implementation was associated with a substantial reduction in malaria infection and illness as well as anaemia in the intervention district. Prevalence of clinical malaria, malaria infection and anaemia were reduced by 80, 65 and 53%, respectively, in the intervention district of Kita compared to the comparison district of Bafoulabe. These reductions in the context of routine programme implementation are consistent with those reported in the clinical trials in Mali and Burkina Faso [5–7, 16]. The choice of the comparison district was appropriate since these indicators were similar in the two districts before intervention. Nevertheless, the possibility of selection bias could not be excluded. To minimize the chance of such bias, a random cluster sampling method was used in both districts, and the sample size estimation and statistical analysis were adjusted for cluster sampling. Analysis of routine data in nine randomly selected health centres in Kita and seven in Bafoulabe also indicated a substantial reduction in the number of cases of uncomplicated and severe malaria. As in most districts in Mali, the routine data in 2013 and 2014 were very incomplete in the intervention and comparison districts. Routine data could better reflect the impact of SMC and other interventions if these data were more complete. As SMC and other malaria control interventions are being scaled up, improving the quality of routine data will be of greater importance.

Coverage of SMC among eligible children was relatively high. Over 80% of children received at least the first day’s SMC treatment irrespective of the method used to assess the coverage (caregiver interview or SMC card) during the first round. However, this proportion decreased over the consecutive rounds and was only 67% for the fourth round based on interviews and 58% based on SMC cards. This emphasizes the importance of continuing to monitor SMC coverage over subsequent years, as well as undertaking activities, including social mobilization and community engagement, to prevent the decrease in coverage seen in subsequent rounds. Travel was reported as the main reason for missing SMC rounds. At the time of this evaluation SMC in the Kayes Region was implemented only in Kita district. With the extension of the SMC to other districts and the plan to cover the whole country in 2016, it is expected that fewer children will miss their rounds because of travel within country. Nevertheless, information and sensitization of communities about the SMC schedule will remain essential to reduce the proportion of children who miss their SMC rounds. After travel, the next most common reason parents reported for missing a round was that they were not informed; efforts should be undertaken to improve information and reminders about the dates for upcoming SMC rounds, for example by providing information at each SMC round, and through reminders from community health workers/other community channels closer to the next round. It is possible that the impact of SMC on malaria and anemia demonstrated in this study could be even greater if coverage of the intervention is improved.

Seasonal malaria chemoprevention was well tolerated with similar or fewer patients reporting side effects than reported in previous clinical trials in Mali [5, 6]. A relatively large proportion of children (21.3%) reportedly spit out (rejected from month) or vomited the first day’s SMC treatment, indicating that a large proportion of replacement doses needs to be considered when planning implementation. The proportion of spitting or vomiting of the replacement day 1 dose was lower (8% on average) and was similar to the proportion of spitting or vomiting of the day 2 and 3 doses administered by parents at home (6.2 and 6.5%, respectively). This difference in proportions of spitting or vomiting on day 1 compared to days 2 and 3 may be due to combination of SP + AQ on day 1 while on days 2 and 3 only AQ was given. This difference could also be due to health workers administering the dose on day 1, versus mothers or caregivers on days 2 and 3. The use of the new dispersible formula with orange flavour recently made available may improve the tolerability and reduce the proportion of the spitting or vomiting.

Not surprisingly, parents’ opinions about SMC were very positive, with 99.9% of parents reporting they felt the strategy is good or very good, and 99% of them supporting the continuation of the intervention. This strong support and favorable opinion of SMC are major assets for continuation and scale-up, and are reflected in the very high rates of administration of drugs for days 2 and 3 by parents at home 95% of the times. Not giving the second and third days treatment, although a major concern, was not an issue according to data from this study, since these treatments were given at least 95% of the time, and only parents of two children reported that they saved the drugs of future treatment or gave it to another child.

The frequency of *dhps* K540E mutation, which serves as a marker for the quintuple mutation that is most associated with the resistance to SP in West Africa [17], remained low (<5%), as did mutations in *Pfmdr1*-86Y; these were similar before and after the intervention in the two districts. This is reassuring despite the increase in dhps A437G mutation given the 50% threshold for *dhps* K540E mutations recommended by WHO for not using SP in intermittent preventive treatment in infants [18]. These data are consistent with what was reported in previous trials in Mali and in Burkina Faso in 2008, with no significant increase in the frequency of these markers at the end of the season in the intervention group compared to the control group after 1 year of implementation [5, 6]. No *dhps* K540E mutations were detected in samples collected after 1, 2 and 3 years of SMC in Senegal [19]. Pilot implementation of intermittent preventive treatment with SP in infants (IPTi) in Mali also did not show an increase of these markers after 1 year in Mali [20]; however, continued surveillance is important, as in Senegal an increase in triple *dhfr* mutants was reported after 2 years of implementation of IPTi [21] and an increase in dhps A437G mutation in post-intervention period in the intervention district is found in this study. Monitoring of molecular markers should become a routine part of the intervention in Mali and other countries implementing SMC.

Strengths of this study include the fact that evaluation was done by a research team not involved with the SMC implementation, the choice of appropriate comparison district, the pre and post design and the assessment of several indicators for SMC implementation. As with any observational study, there is potential for selection and observational bias. To minimize these biases, study participants were selected randomly and technicians who read blood smears or performed the molecular analysis were blinded. Potential for recall bias could not be excluded despite the fact coverage survey was performed only about 4 weeks post SMC rounds and adherence surveys in 4–7 days post SMC rounds.

## Conclusions

In summary, routine implementation of SMC in Mali substantially reduced prevalence and incidence of malaria, as well as prevalence of anemia, with reductions of similar magnitude to those seen in previous clinical trials. Improving coverage through social mobilization and communication could further strengthen SMC impact. One year of implementation of SMC was not associated with an increase in molecular markers known to be associated with resistance to SP and AQ, but resistance markers should continue to be closely monitored.

## Abbreviations

AQ: amodiaquine
DD: difference-in differences
dhfr: dihydrofolate reductase
dhps: dihydropteroate synthetase
Hb: haemoglobin
IPTi: intermittent preventive treatment in infants
MCSP: Maternal and Child Survival Programme of Save the Children
MRTC: Malaria Research and Training Centre
OR: odds ratio
PCR: polymerase chain reaction
Pfcrt: *Plasmodium falciparum* chloroquine transporter gene
Pfmdr1: *Plasmodium falciparum* multidrug resistance gene on
PMI: President’s Malaria Initiative
RCT: randomized controlled trials
RDT: rapid diagnostic test
RFLP: restriction fragment length polymorphism
SMC: seasonal malaria chemoprevention
SP: sulfadoxine–pyrimethamine
USAID: United States of America Agency for International Development
WBC: white blood cells
WHO: World Health Organization
ITN: insecticide-impregnated bed nets
MIS: Malaria Indicator Survey
